# Prognostic Value of CRP/25 OH Vitamin D Ratio for Glucocorticoid Efficacy in Acute Severe Ulcerative Colitis Patients

**DOI:** 10.3390/diagnostics14192222

**Published:** 2024-10-05

**Authors:** Andreja Nikolic, Dragan Popovic, Srdjan Djuranovic, Aleksandra Sokic-Milutinovic, Sanja Dragasevic

**Affiliations:** 1 Clinic for Gastroenterohepatology, University Clinical Center Serbia, Koste Todorovica Street 2, 11000 Belgrade, Serbiadragasevicsanja@gmail.com (S.D.); 2 Faculty of Medicine, University of Belgrade, Dr. Subotica Street 8, 11000 Belgrade, Serbia

**Keywords:** acute severe ulcerative colitis, glucocorticoid response, CRP/25 OH vitamin D ratio

## Abstract

**Introduction**: Acute severe ulcerative colitis (ASUC) represents a life-threatening medical emergency. One-third of ASUC patients are steroid non-responders. Our study aimed to create a new ASUC algorithm to predict corticosteroid response in the early course of the disease. **Materials and Methods:** A cross-sectional study included 103 patients with ASUC (65 male, 38 female). We calculated the serum CRP to 25-hydroxy 25 OH vitamin D ratio at admission. Logistic regression determined patients’ response to glucocorticoids, depending on the CRP/25 OH vitamin D ratio value. **Results and Discussion:** Significant differences were observed in the CRP/25 OH vitamin D ratio at admission between glucocorticoid responders and non-responders (*p* = 0.001). A negative correlation was found between glucocorticoid response and CRP/25 OH vitamin D levels (Spearman’s rho = −0.338, *p* < 0.01). Logistic regression revealed a significant association (*p* = 0.003) with a model chi-square value of 11.131 (*p* = 0.001). ROC curve analysis showed an AUC of 0.696 (*p* = 0.001), indicating moderate discriminatory ability. To achieve 91% sensitivity, the CRP/25 OH vitamin D ratio must be less than 3.985 to predict a complete glucocorticoid response. **Conclusions:** The serum CRP to 25 OH vitamin D ratio on the first day of hospital admission can potentially determine the response to glucocorticoids in patients with ASUC and significantly affect the mortality of these patients.

## 1. Introduction

Ulcerative colitis (UC) is a chronic disease of the large intestine that continuously affects its mucosa, starting from the rectum and moving proximally. The cause is still unknown. However, it is now believed that complex interactions between environmental factors and an aberrant immune response to microbiota components in genetically susceptible individuals lead to the development of UC [1]. The disease course of UC is characterized by phases of remission and relapses. According to extensiveness, UC includes “proctitis”, “left-sided colitis”, and “extensive colitis”, while based on symptoms, signs, and laboratory analyses, the disease can have a “mild”, “moderate”, or “severe” course [2].

The most alarming form is acute severe UC (ASUC) based on Truelove’s and Witts’ criteria (Table 1). The criteria for ASUC are based on ≥6 bloody stools per day and at least one of the following: pulse rate > 90 beats/min, temperature > 37.8 °C, hemoglobin level < 10.5 g/dL, or erythrocyte sedimentation rate > 30 mm/hr [3]. Approximately 25% of UC patients with relapse require in-hospital treatment [3]. During disease exacerbation, correct assessment of the patient’s condition and the extent of the disease determines the subsequent therapeutic algorithm. Biological treatments are often the therapy of choice in current therapeutic strategies. Nevertheless, ongoing collaboration with surgeons and assessments for potential colectomy are necessary for ASUC patients in clinical practice [4].

Predictive scores should be established to indicate diverse therapy modalities in order to determine the therapeutic algorithm in ASUC patients in the early course of the disease. The most used criteria are Travis’, published in 1996 [5]. The criteria of Ho or Scottish [6], Lindgren [7], Seo [8], and Jain [9] are also applied (Table 2), incorporating symptoms, signs, laboratory, and endoscopic findings. The crucial role of the third day in the disease course lies in the assessment and determination of the mentioned parameters [10]. If there is no adequate response to corticosteroids, the patient needs rescue therapy. A colectomy is the last solution, and a late decision on this treatment modality leads to increased postoperative risk and mortality. Therefore, before deciding on rescue therapy, it is necessary to recognize clear surgical indications (Table 3). Rescue therapy involves the use of infliximab, cyclosporin, or tacrolimus. Many factors influence the effectiveness of this therapy, so unique prescribing regimens have been developed for this form of UC. In the case of no response, other medications can be used, although the current data are scarce [11].

Several scores were established in determining the severity of UC activity, including the C-reactive protein (CRP) to albumin ratio [12]. Such models can potentially, in the early stage of relapse, help in the selection of therapy. Also, 25 OH vitamin D, as a modulator of the immune response, has been implicated in the pathogenesis and severity of UC. Emerging research data have determined an association between 25 OH vitamin D deficiency and more severe UC activity.

Our study aimed to develop a simple model that would determine the response to glucocorticoids on the first day of hospitalization. We determined a CRP to 25 OH vitamin D ratio, hypothesizing that the higher the ratio, the lower the chances of glucocorticoid response.

## 2. Materials and Methods

This cross-sectional study encompassed 103 patients (65 male, 38 female) ranging from ages 18 to 47, who required hospitalization for ASUC in the University Clinical Center of Serbia’s Department of Gastroenterology from March 2020 to August 2023. The diagnosis of ASUC was made based on conventional clinical, laboratory, endoscopic, and histopathological evidence [3]. The colonoscopy procedure was performed, enabling biopsy collection and endoscopic evaluation.

Blood samples from ASUC patients were obtained upon hospital admission and analyzed using an enzyme immunoassay for determining the C-reactive protein concentration and liquid chromatography-tandem mass spectrometry (LC-MS/MS) to determine the levels of 25-hydroxy 25 OH vitamin D.

Based on the response to administered glucocorticoids, patients were categorized into two groups based on Travis’ criteria (Table 1) [5]: the first group comprised “complete responders,” while the second group included “incomplete” and “non-responders.” To improve patient analysis, we categorized them into three subgroups: newly diagnosed or inadequately treated (26 patients), mesalazine-only therapy at relapse (52 patients), and non-mesalazine therapy at relapse (26 patients) (Table 4).

This study was conducted in accordance with the Helsinki Declaration. Prior to obtaining written informed consent from each study participant, patients received verbal information about the procedures and the following research. Participants received adequate information about the research purpose and the nature of their participation, giving their consent voluntarily and informedly.

Statistical analyses were performed using SPSS 26.0 (IBM, Armonk, NY, USA) to determine interrelationships of the CRP/25 OH vitamin D ratio upon admission with the glucocorticoid response. Mann–Whitney U tests compared the mean values of the CRP/25 OH vitamin D ratio upon admission between different glucocorticoid response groups. Numeric data were tested for normal distribution using the Kolmogorov–Smirnov test. Logistic regression was used to determine the probability of glucocorticoid response based on this variable. Receiver operating characteristic (ROC) curves were used for the determination of the sensitivity and specificity of the variable’s performance as a diagnostic test for predicting glucocorticoid response. A *p*-value less than 0.05 was considered statistically significant.

## 3. Results

### 3.1. Demographic Characteristics

The current study evaluates a cohort comprising 103 patients with a median age of 42.13 years. Age at inclusion ranged from 18 to 75 years, with a median age at diagnosis of 36.12 years. The composition of the cohort was 62 males and 41 females. Smoking status was categorized as follows: 18 current smokers, 13 former smokers, and 72 non-smokers. The mean symptom duration prior to hospitalization was 43.8 days. The patient’s characteristics are shown in Table 4.

The average duration of hospitalization was 22.98 days, with a spectrum from 7 to 122 days. Glucocorticoid therapy was administered to all patients within this cohort, with 57 classified as ‘complete responders’ to the treatment and 46 exhibiting ‘incomplete’ response or non-response.

The cohort included 15 newly diagnosed patients with ulcerative colitis during the present hospitalization. Before admission, treatment regimens were limited to monotherapy with mesalazine in 52 cases, azathioprine in 5 cases, infliximab in 7 cases, adalimumab in 1 case, tofacitinib in 1 case, and vedolizumab in 11 cases. A subgroup of 11 patients were identified as non-adherent to any regular pre-admission therapy regimen; these individuals were not newly diagnosed cases but rather exhibited irregularity in following their prescribed treatment protocols prior to admission. The mean endoscopic Mayo score (eMayo) at diagnosis was 2.42, which marginally increased to 2.59 at admission. Additional assessment scores recorded included a Travis score of 2.29 [5], Ho score of 2.14 [6], Lindgren score of 1.63 [7], and Seo score at admission of 2.61 [8]. Biochemical markers upon admission indicated a median CRP level of 71.67, with a median 25 OH vitamin D level of 42.94. In terms of disease distribution, proctitis was documented in one patient, left-sided colitis in 23 patients, and extensive colitis in the majority of patients. Upon admission, the cohort exhibited a mean CRP/25 OH vitamin D ratio of 2.087, with observed values ranging from a minimum of 0.03 to a maximum of 11.97 (Table 4).

### 3.2. Prognostic Value of CRP/25 OH Vitamin D Ratio

There were statistically significant differences (*p* = 0.001) in the CRP/25 OH vitamin D ratio at admission between subjects who did and did not respond to glucocorticoids. Spearman’s correlation coefficient shows a significantly negative correlation of −0.338 (*p* < 0.01) between glucocorticoid response and CRP/25 OH vitamin D levels at admission. This suggests that subjects with a positive glucocorticoid response tend to have lower CRP/25 OH vitamin D levels at admission. Logistic regression analysis indicated a statistically significant relationship (*p* = 0.003) between the CRP/25 OH vitamin D ratio at admission and the glucocorticoid response. The model’s chi-square value stands at 11.131 (*p* = 0.001), confirming its statistical significance. ROC curve analysis yielded an area under the curve (AUC) of 0.696 (*p* = 0.001), indicating moderate discriminatory ability for this parameter.

The “newly diagnosed or inadequately treated” subgroup was formed by merging the “de novo” and “without therapy” categories due to sample size considerations. In the newly diagnosed or inadequately treated subgroup, the mean CRP/25 OH vitamin D ratio was 2.7973 (SD = 3.10011). The Mann–Whitney U test showed a statistically significant difference between patients who responded to therapy and those who did not (*p* = 0.004). Spearman’s rho indicated a significant negative correlation (ρ = −0.556, *p* = 0.003), and logistic regression confirmed the score as a significant predictor of response (B = −0.376, *p* = 0.034, Exp (B) = 0.687). ROC analysis demonstrated good discriminative power with an AUC of 0.837, with optimal cutoffs at 5.9500 (sensitivity = 94.1%, specificity = 44.4%), 4.1850 (sensitivity = 82.4%, specificity = 44.4%), and 2.1400 (sensitivity = 76.5%, specificity = 77.8%).

In the mesalazine-only therapy at relapse subgroup, the mean CRP/25 OH vitamin D ratio was 1.7790 (SD = 1.90611). The Mann–Whitney U test revealed a significant difference between responders and non-responders (*p* = 0.026), with Spearman’s rho showing a significant negative correlation (ρ = −0.312, *p* = 0.024). Logistic regression also supported the score’s predictive value (B = −0.375, *p* = 0.033, Exp (B) = 0.687). The ROC analysis indicated moderate discriminative power with an AUC of 0.681, with the best cutoffs being 4.7000 (sensitivity = 96.6%, specificity = 82.6%), 3.9850 (sensitivity = 93.1%, specificity = 73.9%), and 2.4450 (sensitivity = 82.8%, specificity = 60.9%).

In the non-mesalazine therapy at relapse subgroup, the mean CRP/25 OH vitamin D ratio was 1.9154 (SD = 1.55175). The Mann–Whitney U test did not reveal a significant difference between the groups (*p* = 0.297), and correlation analysis found no significant association (ρ = −0.216, *p* = 0.289). Logistic regression did not confirm the score as a significant predictor (B = −0.409, *p* = 0.161, Exp (B) = 0.664). ROC analysis showed moderate discriminative power with an AUC of 0.625, with optimal cutoffs at 2.6600 (sensitivity = 83.3%, specificity = 57.1%), 2.4650 (sensitivity = 75.0%, specificity = 57.1%), and 1.6200 (sensitivity = 66.7%, specificity = 42.9%).

These findings suggest that the CRP/25 OH vitamin D ratio has significant potential as a predictor of glucocorticoid therapy response in the newly diagnosed or inadequately treated and mesalazine-only therapy at relapse subgroups, while its predictive value in the non-mesalazine therapy at relapse subgroup appears more limited and warrants further investigation.

## 4. Discussion

### 4.1. The Role of CRP and 25 OH Vitamin D in the Monitoring and Management of ASUC

UC represents a persistent clinical challenge, primarily since approximately one-quarter of patients encounter relapses necessitating hospitalization. Rapid and accurate diagnosis and therapeutic intervention are crucial, and this research provides significant information in this context [11].

Alongside the established complexities of UC, the importance of biomarkers, with a particular emphasis on CRP, remains a cornerstone in the clinical monitoring of UC patients. Traditionally, endoscopy was the gold standard for confirming mucosal healing (MH) [13,14,15]. However, the advent of biomarkers like CRP offers a rapid and efficient method for patient assessment. Originating from the liver in response to inflammation, CRP has been frequently utilized in evaluating a myriad of inflammatory diseases [16]. Its significance has been particularly underscored in monitoring patients with ASUC. However, the use of CRP in clinical practice is not without its challenges. For instance, although it is adept at evaluating patients in the active phase of IBD, diagnosing MH or a state of inactivity can be challenging when CRP levels are low [17]. Despite CRP being useful in differentiating IBD from non-IBD conditions, many IBD patients, including 71% with ulcerative colitis and 25% with Crohn’s disease (CD), had normal CRP levels at diagnosis. CRP values at diagnosis were higher in patients with CD than those with ulcerative colitis. According to published data, higher CRP levels correlated with an increased risk of surgery in IBD [18]. Genetic factors, age, sex, and body mass index can influence CRP levels. According to a study by Danik et al., genetic variation in the C-reactive protein (CRP) gene affects CRP levels during acute coronary ischemia, with certain haplotypes significantly increasing or decreasing these levels. This is relevant for UC, as CRP is a key inflammatory marker. Understanding genetic factors that influence CRP levels can improve the interpretation of CRP measurements in UC [19]. This highlights the necessity of integrating CRP with other biomarkers or diagnostic methods for a more comprehensive assessment.

The significance of CRP in individuals with ASUC lies in its potential to predict treatment outcomes, leading to its inclusion in the Travis criteria [5]. However, relying solely on CRP is inadequate, especially for evaluating MH. Therefore, it is complemented by clinical evaluations, additional laboratory tests, endoscopic examinations, and radiological assessments of patients.

In addition, 25 OH vitamin D, a fat-soluble vitamin, has garnered attention in recent years for its potential role beyond bone health. Emerging evidence suggests its significance in the context of UC, particularly concerning inflammation and its influence on the gut microbiota.

Briefly, 25 OH vitamin D impacts UC by modulating the immune system. It promotes anti-inflammatory cytokines and inhibits pro-inflammatory T cells, reducing gut inflammation [20,21]. Supplementation significantly increases serum 25 OH vitamin D levels, lowers CRP levels, and enhances calcium absorption, which is crucial for managing UC symptoms [22,23,24]. A meta-analysis by Gubatan et al. confirmed these benefits, showing reduced CRP and erythrocyte sedimentation rate (ESR) levels [10,25].

Furthermore, 25 OH vitamin D also regulates genes involved in immune function and cellular stress response [26]. Higher doses (≥300,000 IU/day) are more effective in achieving clinical improvements, suggesting a dose-dependent effect [27,28]. This is particularly relevant as UC patients often have lower baseline 25 OH vitamin D levels [29,30].

However, variability in study outcomes and limitations such as small sample sizes and short intervention periods necessitate more rigorous research [31,32,33]. Despite these challenges, 25 OH vitamin D shows promise as an adjunct therapy in UC, aiding in inflammation management and maintaining bone health and calcium homeostasis, often compromised in UC patients [34,35].

A pivotal randomized controlled trial by Sharifi et al. [36] delved into the impact of 25 OH vitamin D3 on inflammation and the expression of the cathelicidin gene in UC patients. The findings underscore the potential anti-inflammatory properties of 25 OH vitamin D3 in this patient cohort [37]. Another noteworthy clinical trial by Mathur et al. (2017) centered on the effects of 25 OH vitamin D3 in UC patients diagnosed with hypovitaminosis D3, further emphasizing the vitamin’s therapeutic potential [37].

The gut microbiota, a complex community of microorganisms residing in the intestines, plays a crucial role in health and disease. Furthermore, 25 OH vitamin D has demonstrated a beneficial effect in both CD and UC patients [38]. Specifically, it has been shown to increase the presence of Enterobacteriaceae while concurrently reducing overall intestinal inflammation [38]. In a study administering 25 OH vitamin D at a dose of 40,000 IU weekly over an 8-week period to UC patients, there was a reduction in *Ruminococcus gnavus*. This suggests that 25 OH vitamin D might have a modulating effect on certain bacterial populations within the gut, potentially influencing the overall inflammatory environment in UC patients [39].

The chronic inflammation associated with UC triggers a cascade of immune responses that further damage the intestinal lining. This inflammation can also affect the function of the 25 OH vitamin D receptor (VDR), which is crucial for the cellular uptake and utilization of 25 OH vitamin D. The inflammation and ulceration in UC reduce the absorptive surface area of the intestine, thereby impairing the efficient uptake of 25 OH vitamin D from the diet. Moreover, frequent diarrhea, a common symptom of UC, leads to the rapid transit of intestinal contents, giving less time for the absorption of nutrients [40,41].

Furthermore, 25 OH vitamin D administration was found to attenuate the severity of chemically induced colitis and bolster mucosal healing processes. It is imperative to note that 25 OH vitamin D deficiency in IBD is multifactorial. Factors such as inadequate sun exposure, dietary restrictions, and impaired absorption contribute to this deficiency, which is more pronounced in IBD patients compared to the general population.

According to the results of our study, 25 OH vitamin D levels did not exhibit statistically significant variations based on the four seasons (*p* = 0.172). It is noteworthy to mention that 25 OH vitamin D concentrations can be influenced by a myriad of factors; however, in our cohort, seasonal variations did not play a significant role in altering these levels.

The therapeutic potential of 25 OH vitamin D supplementation in UC has been the focus of several studies. Karimi et al. (2019) embarked on a study exploring the effects of two distinct 25 OH vitamin D regimens on the UC activity index, patient quality of life, and the oxidant/antioxidant balance, and suggested that 25 OH vitamin D supplementation might have a role in modulating disease activity and improving the overall well-being of UC patients [42]. Another intriguing study by Arihiro et al. (2019) conducted a randomized trial assessing 25 OH vitamin D supplementation’s efficacy in preventing seasonal influenza and upper-respiratory infections in IBD patients [43].

Given the individual roles of CRP and 25 OH vitamin D in UC, the CRP/25 OH vitamin D ratio emerges as a potentially significant marker. This ratio encapsulates both the inflammatory status (via CRP) and immune modulation (via 25 OH vitamin D) in UC patients. One of the key findings of this research is the negative correlation between CRP/25 OH vitamin D levels and the response to glucocorticoids. This ratio suggests that patients with higher levels of CRP relative to 25 OH vitamin D are less likely to respond positively to glucocorticoid therapy.

It is important to note that while biologic drugs have become dominant in the therapeutic strategy for UC, surgery remains a significant option for many patients. This research underscores the significance of promptly identifying patients who might not respond to glucocorticoid therapy, facilitating more rapid and effective decisions regarding treatment with biologic agents or surgical intervention.

In an effort to maximize the identification of patients likely to respond favorably to glucocorticoids, a range of cutoff points were explored. The optimal CRP/25 OH vitamin D ratio cutoff values for predicting glucocorticoid response in different patient subgroups are as follows: In the newly diagnosed or inadequately treated subgroup, the optimal cutoff is 2.1400 with a sensitivity of 76.5% and a specificity of 77.8%. In the mesalazine-only therapy at relapse subgroup, the cutoff value of 3.9850 provides a sensitivity of 93.1% and a specificity of 73.9%. In the non-mesalazine therapy at relapse subgroup, the optimal cutoff is 2.6600, with a sensitivity of 83.3% and a specificity of 57.1%. For general application across all patient groups, a cutoff value of 2.7350 (sensitivity: 84.2%, specificity: 43.5%) or 3.9850 (sensitivity: 91.2%, specificity: 32.6%) may be considered, depending on the clinical priority of maximizing sensitivity.

### 4.2. Advantages of CRP/25 OH Vitamin D Ratio over Other Scoring Systems

The CRP/25 OH vitamin D ratio offers several distinct advantages over other scoring systems. Below is a comparison highlighting the key benefits of this ratio in terms of objectivity, predictive value, accessibility, and overall clinical utility.

1. Objectivity and Simplicity: The CRP/25 OH vitamin D ratio is derived solely from laboratory measurements (CRP and 25-hydroxyvitamin D), which minimizes subjectivity in its application. In contrast, other scoring systems like the Ho score and Lindgren score require subjective clinical inputs, such as stool frequency and the presence of blood in the stool, potentially introducing variability and error. Additionally, the Jain score involves endoscopic evaluation of the Ulcerative Colitis Endoscopic Index of Severity (UCEIS) and requires stool samples for fecal calprotectin, which can be uncomfortable for patients during an ASUC episode.
2. Cost-Effectiveness and Accessibility: The CRP/25 OH vitamin D ratio relies on commonly available laboratory tests, making it more accessible and less resource-intensive compared to other scores. For example, the Jain score and Seo score necessitate more sophisticated and costly procedures, such as endoscopy and fecal calprotectin assays, which may not be readily available in all clinical settings, particularly in resource-limited environments.
3. Reduced Risk of Procedural Complications: Unlike other scores that require invasive diagnostic procedures (e.g., endoscopy for the Jain score or radiological assessments for the Ho score), the CRP/25 OH vitamin D ratio is based on a simple blood test, reducing the risk of complications and making it safer for patients, especially those in critical condition.
4. Broad Applicability and Practicality: The CRP/25 OH vitamin D ratio is practical for use in a wide range of healthcare settings, including those with limited resources, because it does not require specialized equipment or advanced training. Its simplicity and ease of calculation make it an efficient tool compared to more complex models like the Seo score and Lindgren score.
5. Integration of Immune Status Monitoring: By incorporating vitamin D, this score not only reflects inflammatory status but also provides insights into the patient’s immune function, which could be crucial for monitoring the response to therapy. This adds a layer of clinical relevance that is not provided by other scores.
6. Consistency and Reliability: The CRP/25 OH vitamin D ratio depends on stable, objective laboratory parameters, reducing the likelihood of errors associated with subjective assessments found in other scores, such as stool count in the Lindgren score or the presence of blood in the Seo score. This enhances the reliability of the score and its clinical utility.
7. Economic and Logistical Considerations: The CRP/25 OH vitamin D ratio is cost-effective and logistically straightforward, as it avoids the need for additional expensive tests or specialized procedures. This makes it a practical choice for various healthcare settings, regardless of resource availability.

### 4.3. Advantages of CRP/25 OH Vitamin D Ratio over CRP and Vitamin D

In the same cohort used to develop the CRP/25 OH vitamin D ratio score, we analyzed the prognostic value of CRP levels on admission, CRP levels on the third day, and vitamin D levels to assess their effectiveness in predicting glucocorticoid response in patients with ASUC. CRP on admission significantly differed between responders and non-responders (*p* = 0.004), but with an AUC of 0.333, its discriminatory power is notably limited. Similarly, CRP on the third day showed a significant difference (*p* = 0.002), yet with an AUC of 0.322, it also demonstrated poor predictive capacity. Vitamin D levels did not differ significantly between groups (*p* = 0.184) and yielded an AUC of 0.424, further underscoring its limited utility as a standalone marker. Logistic regression indicated that neither CRP on admission (*p* = 0.729) nor vitamin D (*p* = 0.396) was a significant predictor. CRP on the third day approached significance (*p* = 0.085) but lacked statistical robustness.

In our analysis, the CRP/25 OH vitamin D ratio outperformed both individual CRP levels and vitamin D levels in predicting glucocorticoid response, providing more reliable and actionable data.

Although CRP is recognized in the literature as a predictor of glucocorticoid response, our findings suggest that it does not exhibit the same level of accuracy in this specific population. This reinforces the utility of the CRP/25 OH vitamin D ratio as a superior tool for early prediction in clinical practice. Furthermore, despite a thorough search on PubMed, we found no studies that specifically measure or address vitamin D levels in ASUC populations. However, it is well documented that vitamin D levels are generally low in patients with ulcerative colitis, highlighting the potential relevance of this marker when combined with CRP in predicting therapeutic outcomes.

### 4.4. Effects of Glucocorticoids on 25 OH Vitamin D

Glucocorticoids have potent anti-inflammatory and immunosuppressive effects and can significantly affect the metabolism of 25 OH vitamin D, the major circulating form of vitamin D. Long-term use of glucocorticoids may exacerbate vitamin D deficiency by impairing its conversion into the active form, calcitriol, and by increasing the metabolism and clearance of 25 (OH)D.

In the context of ASUC, glucocorticoid therapy may have several potential effects on 25 OH vitamin D levels, with potentially beneficial implications. While primarily used to control inflammation and suppress immune responses, glucocorticoids also influence vitamin D metabolism. One benefit of glucocorticoid therapy could be reductions in inflammation, leading to improved gastrointestinal health and enhanced nutrient absorption, including vitamin D, from the gut. Effective control of colitis symptoms could help stabilize or improve vitamin D levels if malabsorption is a factor. Nonetheless, monitoring vitamin D during glucocorticoid therapy remains crucial due to the complex interplay between inflammation control and vitamin D metabolism [44].

## 5. Conclusions

UC is a chronic disease that requires careful and timely diagnosis and therapeutic intervention. This research has identified the potential role of the CRP/25 OH vitamin D ratio as an early indicator of response to glucocorticoid therapy in UC patients. Our results show a negative correlation between the CRP/25 OH vitamin D ratio and therapeutic response, implying that higher CRP/25 OH vitamin D ratios may indicate a lower likelihood of a positive response to glucocorticoid treatment. An added advantage of this ratio is its determination upon admission, rather than waiting until the third day or later during hospitalization. This is particularly significant given that no one has compared this ratio before, making it a potentially important marker in assessing glucocorticoid response. While biologic drugs continue to dominate the therapeutic approaches to UC, such biomarkers can provide valuable information to help clinicians make informed therapy decisions. Further research is needed to confirm these findings and understand their clinical application.

## Figures and Tables

**Table 1:** Disease activity in ulcerative colitis [5].

Variable	Mild	Moderative	Severe
Bloody stools, times/day	<4	4 or more if	≥6 and
Pulse, beats/min	<90	≤90	>90 or
Temperature, C	<37.5	≤37.8	>37.8 or
Hemoglobin, g/L	>115	≥105	<105 or
ESR, mm/hr	<20	≤30	>30
CRP, mg/L	normal	≤30	>30

ESR, erythrocyte sedimentation rate; CRP, C-reactive protein [5].

**Table 2:** Predictive indices of corticosteroid failure in acute severe ulcerative colitis patients and the need for “Rescue Therapy” [2].

Index	Criteria	Predictive Indices
Travis or Oxford criteria [5]	Stool frequency > 8/day or >3/day with CRP > 45 mg/L on 3rd day of IV corticosteroid	If any present on day 3 (85% probability of colectomy)
Ho or Scottish [6]	Colonic dilatation > 5.5 cm (4 points) Albumin < 3 g/dL on admission (1 point) Average daily number of stools across first 3 days: <4 (0 points), 4–6 (1 point), 6–9 (2 points), >9 (4 points)	≥4 points on day 3 (85% probability of nonresponse)
Lindgren [7]	Serum CRP (mg/L) × 0.14 + stool frequency on day 3 of IV corticosteroid	>8 points on day 3 (72% probability of nonresponse)
Seo [8]	The calculated score according to the following variables: hematochezia, stool frequency, ESR, hemoglobin, and albumin	>180 Seo index 2 weeks after determination of corticosteroid probability of colectomy
Jain [9]	A UCEIS > 6 at admission and FC > 1000 µg/g on day 3	Predictors of steroid failure and need for rescue therapy/ colectomy

CRP, C-reactive protein; IV, intravenous; ESR, erythrocyte sedimentation rate; UCEIS, Ulcerative Colitis Endoscopic Index of Severity; FC, fecal calprotectin.

**Table 3:** Indication for surgery in patients who do not respond to rescue therapy [2].

Indication for Surgery in Patients Who Do Not Respond to Rescue Therapy
Toxic Dilatation with Impending Perforation
Intestinal perforation
Massive hemorrhage
Longstanding colitis with “intractability”

**Table 4:** Demographic and clinical characteristics of UC patient cohort.

Characteristic	Data
Total Patients (*n*)	103
Median Age (years, range)	42.13 (18–75)
Gender Distribution (male/female)	62/41
Smokers/Non-smokers	31/72
Mean Symptom Duration Prior to Hospitalization (days)	43.8
Average Hospitalization Duration (days, range)	22.98 (7–122)
Proctocolectomy	1
Glucocorticoid Therapy ResponseGlucocorticoid Incomplete/Non-responders	5746
Previous Glucocorticoid Therapy (*n*)	68
‘De Novo’ UC Patients	15
Pre-Admission Therapy	
Mesalazine	52
Azathioprine	5
Infliximab/Adalimumab/Tofacinib/Vedolizumab	7/1/1/11
Non-Adherent to Pre-Admission Therapy (*n*)	11
Mean eMayo Score at Diagnosis/Admission	2.42/2.59
Additional Assessment Scores	
Travis	2.29
Ho	2.14
Lindgren	1.63
Seo	2.61
Median CRP Level at Admission	71.67
Median 25 OH vitamin D Level at Admission	42.94
Disease Distribution	
Proctitis	1
Left-side colitis	23
Extensive colitis	89
CRP/25 OH vitamin D Ratio at Admission (mean, range)	2.087 (0.003–11.97)

CRP, C-reactive protein; UC, ulcerative colitis.

## Data Availability

The data presented in this study are available on request from the corresponding author due to patient privacy and ethical restrictions.
